# Validation of Genome-Wide Intervertebral Disk Calcification Associations in Dachshund and Further Investigation of the Chromosome 12 Susceptibility Locus

**DOI:** 10.3389/fgene.2012.00225

**Published:** 2012-11-01

**Authors:** Mette Sloth Mogensen, Karsten Scheibye-Alsing, Peter Karlskov-Mortensen, Helle Friis Proschowsky, Vibeke Frøkjær Jensen, Mads Bak, Niels Tommerup, Haja N. Kadarmideen, Merete Fredholm

**Affiliations:** ^1^Faculty of Health and Medical Sciences, University of CopenhagenCopenhagen, Denmark; ^2^National Food Institute, Technical University of DenmarkLyngby, Denmark; ^3^Faculty of Health Sciences, Department of Cellular and Molecular Medicine, Wilhelm Johannsen Centre for Functional Genome Research, University of CopenhagenCopenhagen N, Denmark

**Keywords:** canine, intervertebral disk calcification, LD pattern, haplotype effects, resequencing

## Abstract

Herniation of the intervertebral disk is a common cause of neurological dysfunction in the dog, particularly in the Dachshund. Using the Illumina CanineHD BeadChip, we have previously identified a major locus on canine chromosome 12 nucleotide positions 36,750,205–38,524,449 that strongly associates with intervertebral disk calcification in Danish wire-haired Dachshunds. In this study, targeted resequencing identified two synonymous variants in *MB21D1* and one in the 5′-untranslated region of *KCNQ5* that associates with intervertebral disk calcification in an independent sample of wire-haired Dachshunds. Haploview identified seven linkage disequilibrium blocks across the disease-associated region. The effect of haplotype windows on disk calcification shows that all haplotype windows are significantly associated with disk calcification. However, our predictions imply that the causal variant(s) are most likely to be found between nucleotide 36,750,205–37,494,845 as this region explains the highest proportion of variance in the dataset. Finally, we develop a risk prediction model for wire-haired Dachshunds. We validated the association of the chromosome 12 locus with disk calcification in an independent sample of wire-haired Dachshunds and identify potential risk variants. Additionally, we estimated haplotype effects and set up a model for prediction of disk calcifications in wire-haired Dachshunds based on genotype data. This genetic prediction model may prove useful in selection of breeding animals in future breeding programs.

## Introduction

In the dog, herniation of the intervertebral disk is a common cause of neurological dysfunction. Especially the Dachshund is predisposed with a relative risk 10–12 times higher than all other breeds (Priester, 1976; Goggin et al., 2000) and an estimated lifetime occurrence of 19% (Ball et al., 1982). The intervertebral disks lie between adjacent vertebrae in the vertebral column forming cartilaginous joints that allow slight movements between vertebrae. The disks are complex structures consisting of a gelatinous core called the nucleus pulposus, an outer fibrous ring called the annulus fibrosus, and the cartilaginous endplates representing the cranial and caudal boundaries of the intervertebral disk. In the Dachshund and other hypochondroplastic breeds the predisposition to intervertebral disk herniation is the result of an early degenerative process, which can result in disk calcification (Hansen, 1952). The degeneration is preceded by early chondroid metaplasia emerging from the perinuclear zone and affecting the majority of the nucleus pulposus and perinuclear annulus fibrosus with profound matrix changes occurring within the first year of life (Hansen, 1952; Ghosh et al., 1976). Dogs with several disk calcifications are at particular high risk of herniation, while herniation rarely occurs in dogs without disk calcifications (Stigen, 1996; Lappalainen et al., 2001). A radiographic evaluation of the number of calcified disks at 2 years of age is a good indicator for the severity of the degeneration and associates strongly with the occurrence of clinical disk herniation at a later age (Jensen et al., 2008). The severity of disk degeneration among breeds describes a continuous spectrum suggesting a multifactorial etiology involving the cumulative effects of several genes and environmental factors (Ball et al., 1982). Severe disk degeneration with calcification has previously been shown highly heritable in Dachshund with heritability estimates of 0.47–0.87 (Jensen and Christensen, 2000). To decrease the occurrence of clinical disk herniation in the Danish Dachshund population the Danish Dachshund Club (DDC) has established breeding guidelines. Based on radiographic examinations at 24–42 months of age the number of calcified disks is determined and since 2008, DDC has recommended excluding dogs with ≥5 calcified disks from breeding. Since 2009, screening of breeding dogs has been mandatory and breeding values of disk calcification have been estimated, using a BLUP (Best Linear Unbiased Prediction) Animal model.

Within the past few years genome-wide association studies (GWAS) have identified numerous promising signals of association between genetic variants and human traits. The use of high density SNP arrays have also shown strength in disease mapping in dogs and has opened doors toward a greater understanding of the genetic architecture of several complex diseases (Wood et al., 2009; Wilbe et al., 2010; Madsen et al., 2011). The genetic homogeneity existing within dog breeds and the spontaneous occurrence of specific diseases in different breeds indicate a breed specific accumulation of disease causing genetic factors. This provides the dog with some advantages in studying genetic diseases as fewer markers and individuals are needed when compared with human studies (Sutter et al., 2004; Lindblad-Toh et al., 2005). The association signals identified through GWAS most likely represents only markers of putative risk and not the causal variant itself. Therefore, to generate hypothesis about mechanisms underlying a specific phenotype it is important to identify the causal variants themselves. This is often a difficult task and requires extensive efforts. The dog provides an excellent model to study complex diseases through the use of GWAS due to the extensive LD and long haplotype blocks characteristic of single dog breeds. However, because of long ranging LD in the dog genome, disease-associated haplotype blocks are often large, hampering the identification of the causal variant. Consequently, while the high extent of LD existing in the dog population is an advantage in the initial GWAS it may complicate the subsequent identification of the causative variant(s) (Sutter et al., 2004).

To investigate the underlying genetic mechanisms behind disk calcification, blood samples from Danish Dachshunds were collected through collaboration with the DDC. Previously, based on a GWAS in 33 cases and 28 controls using the Illumina CanineHD BeadChip, we identified a major locus associating with intervertebral disk calcification in wire-haired Dachshunds on a genome-wide level on canine chromosome (CFA) 12 nucleotide positions 36,750,205–38,524,449. We discovered 36 markers within the genomic region with *p*-values between 0.00001 and 0.026 after correcting raw *p*-values for multiple testing by permutation. This provided clear evidence of the region harboring genetic components affecting the development of disk calcification and thus the risk of disk herniation in wire-haired Dachshunds (Mogensen et al., 2011). The associated locus however requires additional exploration to refine the location of the causal variant(s).

This study was performed within the LUPA project (LUPA)^1^ to validate the original GWAS finding and characterize the CFA12: 36,750,205–38,524,449 susceptibility locus. Targeted resequencing was performed to identify potential functional SNPs that could explain the association signal and the local LD pattern across the disease-associated region was defined. Furthermore, haplotype window effects on disk calcification were estimated, to pinpoint a sub region more likely to harbor the causal variant(s).

## Results

The disease-associated region contains a total of seven annotated protein coding genes in Ensembl (version 66.2); *RIMS1*, *KCNQ5*, *DPPA5*, *C6orf221*, *OOEP_CANFA*, *DDX43*, and *MB21D1*. Furthermore, the region harbors a number of non-coding RNAs (ncRNAs): cfa-mir-30c-2, cfa-mir-30a as well as three novel ncRNAs. As none of these genes or ncRNAs have previously been known to influence disk calcification resequencing was used to generate a list of potential mutations that could explain the association signal. Using the NimbleGen Sequence Capture technology and the Illumina platform we enriched and sequenced the target region in one affected and one unaffected dog of wire-hair. A summary of the statistics describing the resequencing data is given in Table 1. Enrichment of the selected genomic region resulted in 631 and 356 fold enrichment for the affected and unaffected sample, respectively, compared to the non-enriched library. A high coverage was achieved for both samples with >96% of the target region being covered by at least one read and >70% of the reads mapping uniquely to the target region.

**Table 1:** **Resequencing statistics**.

	Affected	Unaffected
Average fold enrichment	631	356
Total reads	26,515,913	31,995,941
Uniquely mapped reads	19,007,898	23,112,589
Percent of target region covered by 1+ reads	96.5	96.8
Percent of target region covered by 10+ reads	94.8	95.1
Mean per base coverage	529	648

*Average fold enrichment: the PCR efficiency raised to the power of delta-crossing threshold value (delta-*C*_t_). Total reads: the total number of reads. Uniquely mapped reads: reads were aligned to the target region CFA12: 36,702,118–38,574,449 on CanFam2.0 via Bowtie using default parameters. Percent of target region covered by 1+ or 10+ reads: percent of target bases covered by at least one or 10 reads. Mean per base coverage: average number of reads per base*.

Using the MAQ software (Li et al., 2008) to infer variants from the alignment, we identified 4119 SNPs and 377 indels in the affected dog and 2956 SNPs and 250 indels in the unaffected dog compared to the reference sequence (CanFam2.0) for the domestic dog (*Canis familiaris*; female boxer). The case was homozygous for three SNPs in protein coding regions or untranslated regions (UTRs): two synonymous SNPs in *MB21D1* and one SNP in the 5′-UTR of *KCNQ5*, see Table 2. These three variants where selected for genotyping in 56 unaffected and 28 affected wire-haired dogs of standard size. All three variants were found to associate with disk calcification with the SNP in the 5′UTR of *KCNQ5* showing the strongest association with a *p*-value of 1.4 × 10^−7^, see Table 3. A list of the predicted functional effect on disk calcification for SNPs identified during resequencing can be found in Table A1 in Appendix. By genotyping the three SNPs in a sample of long- and smooth-haired Dachshund, we found no association to disk calcification, data not shown. Instead dogs of these two hair-varieties seem to be fixed for the genotype of affected wire-haired dogs.

**Table 2:** **SNPs in protein coding regions and UTRs for which the case is homozygous**.

SNP position	Gene involved	Type of SNP	Genotype Case/Control	Sequencing reads covering the SNP (case/control)
37,871,992	*KCNQ5*	5′UTR	GG/CC	(291/371)
38,513,135	*MB21D1*	Synonymous	CC/TT	(364/696)
38,514,745	*MB21D1*	Synonymous	TT/AA	(1043/1158)

*SNP position is according to Ensembl *Canis familiaris* version 64.2*.

**Table 3:** **Test of association between SNPs and disc calcification**.

Location	Gene		Genotypes and observed frequencies			χ^2^	*p*-value
37,871,992	*KCNQ5*		CC	GC	GG		
		Controls	7 (0,125)	37 (0,661)	12 (0,214)	31,575	1.4 × 10^−7^
		Cases	1 (0,036)	3 (0,107)	24 (0,857)	
38,513,135	*MB21D1*		TT	TC	CC		
		Controls	5 (0,090)	27 (0,482)	24 (0,428)	14,100	0,00087
		Cases	1 (0,036)	3 (0,107)	24 (0,857)	
38,514,745	*MB21D1*		AA	AT	TT		
		Controls	5 (0,090)	18 (0,321)	33 (0,589)	8,141	0,01707
		Cases	1 (0,036)	2 (0,071)	25 (0,893)	

**Figure 1:**
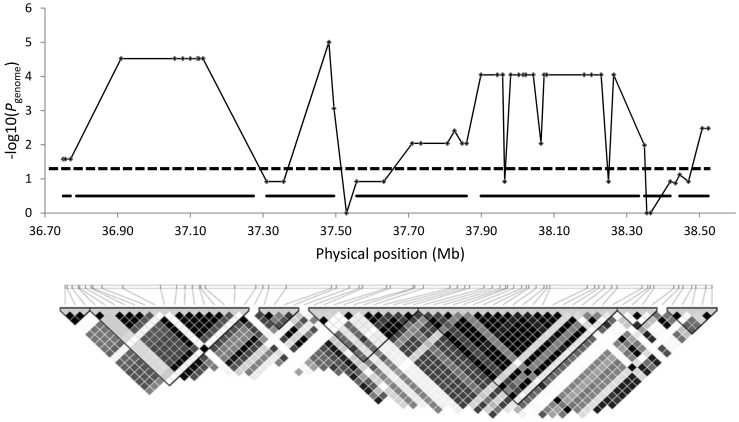
**Association and LD block analysis of the CFA12: 36,750,205–38,524,449 susceptibility locus in wire-haired Dachshunds**. Detailed view of the CFA12 genomic region associating with disk calcification in wire-haired Dachshunds. The *x*-axis show the position on CFA12 in mega bases (Mb) and the *p*-values on the *y*-axis correspond to the *p*-values from the GWAS in wire-haired dogs corrected for multiple testing (Mogensen et al., 2011), as seen in Table A4 in Appendix. The horizontal dotted line represents the threshold of genome-wide significance. The graphical representation of the LD pattern across the region is generated in Haploview 4.2. LD is specified using the *r*^2^-color scheme: *r*^2^ = 0: white; 0 < *r*^2^ < 1: shades of gray; *r*^2^ = 1: black. The black horizontal lines in the Manhatten plot correspond to the position of the LD blocks defined in Haploview.

The ∼1.8-Mb genomic region on CFA12 associating with disk calcification in Danish wire-haired Dachshund (Mogensen et al., 2011) encompass seven LD blocks identified using the four gamete rule (Wang et al., 2002) in Haploview, see Figure 1. The LD blocks range from 20 to 487 kb in size and all blocks include one or more markers significantly associating with disk calcification on a genome-wide level. The marker with the lowest *p*-value corrected for multiple testing (*P*_genome_ = 0.00001) is located at nucleotide position 37,480,959 in LD block 3, which spans 185 kb in size.

Linear and logistic regression analyses were performed to investigate the effect of the haplotypes within each window on disk calcification. The maximal number of haplotypes is 2*^n^*, where *n* is the number of SNPs in a window, which mean that 16 haplotypes could be expected in a four-SNP window. However, with the dataset available and the high extent of LD the observed haplotypes for each of the nine haplotype windows ranged from two to four. The overall significance of which haplotype window explained more genetic variation than the other windows were assessed by the coefficient of determination (*R*^2^), which provides a measure of how well the haplotype effects fitted in the model predicts the disease outcome (case/control) for a particular dog. In generalized linear model (GLM), residual mean deviance (RMD) was used as an indicator for variance explained by the haplotype window and thus the lower the RMD the better is the model fit. Looking at both the linear model and GLM all haplotype windows are significantly associated with disk calcification; see Table 4. Of the nine haplotype windows, we have identified haplotype window 3 as explaining the highest proportion of variance in the disk calcification dataset followed by haplotype window 1 and 2. Haplotype window 3 CFA12: 37,123,193–37,494,845 covers a part of LD block 2 and the entire LD block 3 identified in haploview. Test of association with disk calcification for particular haplotypes within the different haplotype windows, based on both the linear model and GLM are given in Table A2 in Appendix.

**Table 4:** **Haplotype substitution effects for disc calcification scored as binary cases/control disc scores**.

Haplotype window	Nucleotide position on CFA12	Linear model (%)	*p*-value	Logistic model	*p*-value
Hap 1	36,750,205–36,909,311	^¤^*R*^2^ = 73	<0.001	*RMD = 0.64	<0.001
Hap 2	37,056,901–37,119,065	*R*^2^ = 73	<0.001	RMD = 0.64	<0.001
Hap 3	37,123,193–37,494,845	*R*^2^ = 76	<0.001	RMD = 0.46	<0.001
Hap 4	37,710,073–37,826,314	*R*^2^ = 51	<0.001	RMD = 0.92	<0.001
Hap 5	37,847,222–37,944,067	*R*^2^ = 68	<0.001	RMD = 0.75	<0.001
Hap 6	37,958,884–38,015,502	*R*^2^ = 63	<0.001	RMD = 0.85	<0.001
Hap 7	38,022,379–38,072,703	*R*^2^ = 63	<0.001	RMD = 0.82	<0.001
Hap 8	38,079,788–38,229,535	*R*^2^ = 63	<0.001	RMD = 0.85	<0.001
Hap 9	38,264,121–38,524,449	*R*^2^ = 62	<0.001	RMD = 0.76	<0.001

^¤^*R*^2^ = Percent variability in the data set accounted for by the fitted haplotype window model *RMD, Residual mean deviance is an indicator for goodness of fit (the lower the RMD, the better is the model fit).

Based on these analysis we are able to set up a genetic predictions model for disk calcifications ${ŷ}_{i}$ in Dachshunds of the wire-haired variety given their haplotype or genotype information;

(1) $${ŷ}_{i}={\hat{\alpha }}_{0}+{Ŝ}_{i}+\overset{p}{\underset{J=1}{\sum }}{\hat{\alpha }}_{J}.cH_{iJ}$$

where, ${ŷ}_{i}$ is the predicted disk calcification for individual *i*, ${\hat{\alpha }}_{o}$ is the intercept, ${Ŝ}_{i}$ is the estimated sex effect for the *i*^th^ individual, and ${\hat{\alpha }}_{i}$ ${\hat{\alpha }}_{i}$ is the estimated effect for haplotype $H_{ij}$ for *i*^th^ individual with haplotype *J*. Individuals with the least common haplotype were assigned the reference level ${\hat{\alpha }}_{o}$.

## Discussion

We have previously shown that the CFA12: 36,750,205–38,524,449 genomic region associates with disk calcification in wire-haired Dachshund on a genome-wide level (Mogensen et al., 2011). However, a comprehensive study of sequence variation within the region is required to identify the causal variant(s) that might explain the association signal. In this study we have investigated genetic variation within the target region through targeted resequencing in order to identify potential risk variants and validate original GWAS findings. To further investigate the locus we have identified LD block pattern across the disease-associated region and estimated the genetic variation explained by the different haplotype windows. Finally, we have developed a risk prediction model for wire-haired Dachshunds, using the disk calcification and haplotype dataset.

Functional SNPs may have variable effect on protein sequence, transcriptional regulation, splicing, microRNA- and transcription factor binding sites depending on their position and flanking sequences. By targeted resequencing we have made a comprehensive list of potential causal variants that could explain the association signal. A ranking of these SNPs is necessary for follow-up studies to be possible. Numerous SNPs, identified in this study, are predicted to be located within transcription factor binding sites or microRNA-binding sites. Due to the high number of cases sharing the same haplotype we have focused on variants within protein coding regions or UTRs for which the case is homozygous. We have validated the association of one variant in the UTR of *KCNQ5* and two synonymous variants in *MB21D1* in an independent sample of wire-haired Dachshunds hereby confirming the original GWAS and thus providing further evidence for the association of this region with disk calcification. Disk herniation is also seen in long- and smooth-haired Dachshunds. However, interestingly, both cases and controls within these two hair variants appear to be fixed for the haplotype found in wire-haired cases. Thus, presumably other loci must be involved in the development of the disease in long- and smooth-haired variants. This hypothesis is supported by the fact that when 18 controls and 15 cases of long- and smooth-hair were included in our original GWAS (Mogensen et al., 2011), an additional locus, not appearing when including only wire-haired dogs, was detected on CFA3. However, more dogs are needed to confirm this hypothesis. In terms of SNPs validated in the wire-haired dogs any of the three variants may have a potential functional impact on the phenotype in wire-haired dogs. However, it is more likely that these SNPs are markers in high LD with the actual causal variant(s). Resequencing of the target region in a larger number of affected and unaffected dogs might be necessary to eliminate some of the identified variants before a thorough follow-up on other highly ranked variants can be carried out.

To characterize the CFA12 locus and potentially narrow down the candidate region we looked at the LD block pattern. Haploview identify seven LD blocks across the region associating with disk calcification. Several of the markers showing genome-wide significance are in strong LD (*r*^2^ > 0.8) with genome-wide significant markers in other LD blocks indicating the presence of strong LD within the disease-associated region. That this genomic region falls into a segment of strong LD is further documented by 28 of the 33 cases in the GWAS sharing the same haplotype across all 36 genome-wide significant markers within this region (Mogensen et al., 2011). In addition several of the markers show more or less equivalent evidence of association for the given signal indicating that the markers are highly correlated. Given the high extent of LD within this region it is difficult to resolve whether two or more independent loci contribute independent effects to disk calcification.

Analyzing haplotype window effects could potentially pinpoint a haplotype window with a higher effect on disk calcification and thus define or narrow down the region of interest. By estimating the effect of the haplotype windows we have identified window 3 CFA12: 37,123,193–37,494,845 as explaining the largest part of the genetic variation between dogs in our dataset (76%) followed by haplotype window 1 and 2 explaining 73% of the genetic variation. From these results it therefore seems most likely that the causal genetic variant(s) are to be found within the CFA12: 36,750,205–37,494,845 genomic region, which harbors the ncRNAs cfa-mir-30c-2 and cfa-mir-30a as well as a part of *RIMS1*. However, all haplotype windows explain a fair proportion of the variance in the dataset, which is not surprising due to the large amount of LD within this region. Therefore one needs to be careful when narrowing down the region to these three haplotype windows.

A genetic prediction model for intervertebral disk calcification based on these haplotype effects analyses may form a valuable tool for genetic counseling in the wire-haired Dachshund population.

Genome-wide association studies has to a large extent focused on the detection of effects attributable to common SNPs. Other sequence variants such as rarer variants (MAF of 1–5%) and structural variants are also expected to contribute to the genetic basis of common disease and efforts to detect these genetic variations should be included in future studies. Even when a true causal variant is identified challenges remain in reconstructing the molecular mechanisms whereby the variant have an impact on the phenotype of interest and even more work is necessary in translating these findings into advantages in clinical care. Based on a literature search no genes with a direct biological link is present within the disease-associated region one could speculate whether the region contains a regulatory element controlling the expression levels of a causal gene located either upstream or downstream of the candidate region identified here. One hypothesis is a regulatory variant affecting the expression level of *COL9A1*. This gene is located ∼1 Mb upstream of the disease-associated region and encodes one of the three alpha chains of collagen IX. Collagen IX serves as a minor component in the annulus fibrosus and the nucleus pulposus and is thought to be involved in maintaining network integrity in the normal disk. Mutations in *COL9A2* and *COL9A3* have previously been linked to human disk disease (Annunen et al., 1999; Paassilta et al., 2001) and studies in transgenic mice have further demonstrated that mutations in collagen IX can lead to disk degeneration but also degenerative joint disease (Kimura et al., 1996).

## Conclusion

In the present study we validate the previously identified association of the locus CFA12: 36,750,205–38,524,449 with disk calcification in an independent sample of wire-haired Dachshund thus providing strong evidence that variation within this locus affect the development of disk calcification in wire-haired Dachshunds. Moreover, our results suggest that the locus falls within a region of strong LD hence complicating the identification of the causal variant. Our predictions on the effect of the nine different haplotype windows on disk calcification imply that the causal variant(s) are to be found within the CFA12: 36,750,205–37,494,845 genomic region, however care must be taken when drawing this conclusion as all haplotype windows explain a reasonable part of the variability in the disk calcification dataset.

## Materials and Methods

### Animals and diagnostic procedures

This study was confined to Dachshund registered in the DDC. All blood samples included in this study were collected by licensed veterinarians with owners’ consent. Inclusion criteria for sampling were based on radiographic examinations of intervertebral disk calcifications from the second cervical vertebra to the third sacral bone at age 24–42 months (Jensen and Ersbøll, 2000). Information regarding size (standard, miniature, and rabbit), hair variant (wire-haired, long-haired and smooth-haired) sex, age, and pedigree records were obtained from the Danish Kennel Club registry. Disease status of cases and controls were scored based on standard protocol for radiographic examinations; cases were classified as dogs with either ≥6 disk calcifications or dogs that had undergone surgical treatment for disk herniations. Controls were classified as dogs with ≤1 disk calcification. For further information on the distribution of disk calcifications among cases and controls (see Mogensen et al., 2011).

### NimbleGen sequence capture array design and data analyses

For targeted resequencing one affected and one unaffected dog was selected. The affected dog had 12 disk calcifications as evaluated from the radiographic examination and was homozygous across the 36 significantly associated markers in the disease-associated region. The unaffected dog had no disk calcifications and was homozygous for the opposite alleles of the affected dog across the entire region. Both were female standard wire-haired dogs and unrelated at great grandparental level. A custom tiling NimbleGen 385K sequence capture array targeting CFA12: 36,702,118–38,574,449 on CanFam2.0 was designed and manufactured by Roche NimbleGen, Madison, WI, USA. The probe set design was approved with the fraction of bases in the target region covered by probes being 96.5%. Genomic DNA was captured following the NimbleGen Sequence Capture protocol (Roche NimbleGen, Madison, WI, USA). In brief, 25 μg genomic DNA was fragmentized by sonication to blunt-ended fragments and hybridized to the custom array. Unbound fragments were washed away. The target-enriched pool was eluted and recovered from the array and amplified by ligation-mediated PCR. Quantitative fluorescence PCR (qPCR) was performed on pre- and post-enriched libraries to calculate relative-fold enrichment of the targeted region. A locus within the target region was selected for qPCR enrichment analysis with the Stratagene Mx3000P qPCR system using the following primers designed using Primer-BLAST (Primer BLAST)^2^: F: 5′-TGCCTCTGTTGTCCACAGTCAGA-3′; R: 5′-TGCTTGGGGACCTCCTGTCACC-3′. One microgram of captured libraries were subsequently sequenced on the Illumina Genome Analyzer platform as paired end 2 × 36 sequencing reads following the Genome Analyzer User Guide. Bowtie (Langmead et al., 2009) was used to align short read sequence data against the CanFam2.0 reference genome and sequence variants were identified running MAQ (Li et al., 2008) on the reads aligning uniquely to the region.

All SNPs identified from resequencing were evaluated according to their potential functional effect on disk calcification. The SNPs were compared to Ensembl *Canis familiaris* version 64.2 annotations and predictions and SNPs in protein coding regions or within or near predicted ncRNAs were identified. Further SNPs were evaluated based on a measure of conservation in dog, human, mouse, and rat, position according to transcription start site and end site and if the SNP was likely to change the predicted binding of transcription factors or predicted ncRNAs.

### Validation of GWAS findings using TaqMan^®^ SNP genotyping assays

Three SNPs at nucleotide position 37,871,992, 38,513,135 and 38,514,745 were genotyped using Custom TaqMan^®^ SNP Genotyping assays (Applied Biosystems, Foster City, CA, USA) in an independent sample of wire-haired dogs not included in the original GWAS. The sample included 56 controls and 28 cases that had undergone a thorough radiographic examination to determine affection status as descried previously. The primers and probes obtained from the ABI assay kit are specified in Table A3 in Appendix. Reactions were carried out according to the manufacturer’s protocol. Briefly, PCR was performed in the presence of 10 ng genomic DNA, TaqMan^®^ Universal PCR Master Mix, and the SNP Genotyping Assay specific for each SNP. The thermal cycling conditions on Mx3000P^™^ (Strategene) were 95°C for 10 min, followed by 60 cycles at 92°C for 15 s and 60°C for 1 min. Results were analyzed using the MxPro software and the SNPs were tested for genotypic associations with disk calcification using chi-square test statistics.

### Analysis of LD pattern in Haploview

The LD pattern of all 117 SNPs covering the CFA12: 36,750,205–38,524,449 genomic region were analyzed in Haploview 4.2 (Barrett et al., 2005) using SNP genotyping data from the original GWAS with 33 wire-haired cases and 28 wire-haired controls. The four gamete test (Wang et al., 2002) implemented in Haploview using default parameters were used to define the LD block structure and create a graphical representation of the LD pattern. The level of LD is represented by *r*^2^-values.

### Estimation of haplotype effects on disk calcification

The effect of haplotypes in nine haplotype windows was estimated using data from our previous GWAS on disk calcification (Mogensen et al., 2011). The 30 cases and 23 controls included in the analyses were all of standard size and wire-haired to keep the population as genetically homogeneous as possible. For the 36 genome-wide significant markers within the CFA12: 36,750,205–38,524,449 genomic region we defined haplotype windows with four-SNPs creating nine haplotype windows, see Table A4 in Appendix. The haplotype frequencies and most likely haplotype pair (linkage phase) for each dog were estimated from genotyping data using PHASE v.2.1.1 (Stephens et al., 2001). Since haplotypes are reconstructed from genotype data, there are always two haplotypes per dog for each haplotype window. From the PHASE data each dog was assigned a score of 0, 1, or 2 corresponding to 0 copies, 1 copy, or 2 copies of a given haplotype in a haplotype window. Using this haplotype count data, we estimated the effect of each window on disk calcification in dogs. Preparation of data files and methods used for estimating haplotype substitution effects were according to those described for allele substitution models by Kadarmideen et al. (2011) and Kadarmideen (2008). Estimations of haplotype effects on disk calcification was done on a binary scale as cases = 1 (classified as dogs with ≥6 disk calcifications) and controls = 0 (classified as dogs with 0 or 1 disk calcification). Information on sex was included as fixed effects. All analyses were performed in ASReml 3.0 (Gilmour et al., 2002). Linear and logistic regression models were fitted to binary case/control scores on disk calcification. A standard linear haplotype substitution model was:

(2) $$y_{i}=\alpha_{0}+S_{i}+\overset{p}{\underset{J=1}{\sum }}\alpha_{J}.cH_{iJ}+\varepsilon_{i}$$

where, for individual *i*, α_0_ is the intercept, *S_i_* is the sex, and ε*_i_* is the residual. The term *cH_iJ_* is the number of copies (0, 1, or 2) of haplotype *J* (1 to *p*). The least common haplotype was set as a reference level (=α_0_) and the effect of the other haplotypes represents the relative haplotype effect compared to this reference level.

To take the binomial distribution of case/control data we fitted a GLM using the *logit* link function. The model took the following form:

(3) $$\log\left(\frac{\pi_{i}}{1-\pi_{i}}\right)=\alpha_{0}+S_{i}+\overset{p}{\underset{j=1}{\sum }}\alpha_{J}.cH_{iJ}$$

where $\pi_{i}$ is the probability of observing a case $y_{i}=1$ $1-\pi_{i}$ is the of probability of observing a control $y_{i}=0$. All analyses were conducted for each haplotype window one at a time. Significance of the model terms was assessed by *F*-test statistics and associated *p*-values for each haplotype in each haplotype window and other fixed effects. For the linear model (2), the overall model fit for a particular haplotype window was assessed by *R*^2^ values expressed as percentage. This explains the proportion of variance in disk calcification explained by the corresponding haplotype window. Since there is no equivalent expression for *R*^2^ in the GLM framework, the logistic model fit was assessed by the RMD. The RMD represent residual effects not explained by the model; hence the lower the RMD the better is the model fit. For both the linear model and GLM, the overall statistical significance was assessed by *p*-values. It should be noted that linear models (2) were applied to binary case/control data as if they were normally distributed. It has been shown that linear models are quite robust to violation of normality in gene or QTL mapping and association studies and that it is simple to apply and interpret in many studies (Kadarmideen et al., 2000). However, we also applied statistically appropriate GLM to case/control binary data.

## Conflict of Interest Statement

The authors declare that the research was conducted in the absence of any commercial or financial relationships that could be construed as a potential conflict of interest.

## Footnotes

^1^ http://www.eurolupa.org/

^2^ http://ncbi.nlm.nih.gov/tools/primer-blast

## Appendix

**Table A1:** **Functional prediction of SNPs homozygous in the case sorted according to genomic position**.

TaqMan SNPs^a^	Position^b^	Case geno^c^	Cons score^d^	Comments^e^
	36750513	GG	10	TSS PROXIMITY miRNA change TFBS change
	36751290	GG	19	TSS PROXIMITY
	36751590	TT	55	TSS PROXIMITY
	36753585	GG	16	TSS PROXIMITY miRNA change TFBS change
	36762341	TT	41	TSS PROXIMITY miRNA change
	36766398	CC	43	TSS PROXIMITY
	36801723	CC	23	TSS PROXIMITY
	36803655	CC	16	TFBS change
	36809769	CC	62	ncRNA PROXIMITY
	36810002	TT	27	TFBS change
	36814590	CC	58	TFBS change
	36823331	TT	12	miRNA change TFBS change
	36823332	GG	12	miRNA change TFBS change
	36824279	AA	14	miRNA change TFBS change
	36825704	AA	19	TFBS change
	36826666	CC	17	TFBS change
	36826888	CC	23	miRNA change
	36827844	GG	44	miRNA change TFBS change
	36840333	TT	23	TFBS change
	36846574	CC	14	miRNA change
	36847384	TT	13	TFBS change
	36850155	CC	12	TFBS change
	36853046	GG	13	miRNA change
	36853059	GG	14	TFBS change
	36853122	CC	16	miRNA change
	36860038	TT	16	miRNA change
	36871633	AA	11	miRNA change
	36886442	AA	14	miRNA change
	36887486	GG	12	miRNA change TFBS change
	36897624	AA	12	miRNA change
	36899830	AA	27	TFBS change
	36899834	AA	32	TFBS change
	36903284	AA	12	miRNA change TFBS change
	36904787	CC	20	TFBS change
	36906286	TT	18	miRNA change
	36925990	AA	13	TFBS change ncRNA PROXIMITY
	36926238	TT	49	TFBS change
	36928655	CC	17	TFBS change
	36933548	AA	26	miRNA change
	36939600	AA	10	miRNA change
	36941861	AA	14	miRNA change
	36943148	CC	16	miRNA change
	36943511	GG	25	TFBS change
	36951069	GG	11	miRNA change
	36955971	AA	64	TFBS change
	36962236	GG	11	miRNA change
	36962563	CC	51	miRNA change
	36964810	CC	71	TFBS change
	36965584	AA	11	TFBS change
	36965676	GG	12	miRNA change
	36966489	CC	29	TFBS change
	36966628	CC	32	miRNA change TFBS change
	36968474	AA	15	miRNA change
	36971526	AA	11	miRNA change
	36985376	CC	30	miRNA change
	36985633	GG	14	miRNA change
	37025881	CC	11	TSS PROXIMITY
	37028436	CC	49	TSS PROXIMITY
	37059275	CC	13	miRNA change
	37063231	GG	26	TFBS change
	37078527	GG	21	miRNA change TFBS change ncRNA prediction
	37086384	AA	12	miRNA change
	37090854	AA	26	miRNA change
	37095837	CC	47	TFBS change
	37099752	AA	40	miRNA change
	37104620	AA	16	miRNA change
	37104814	TT	25	TFBS change
	37120008	CC	15	miRNA change
	37120009	CC	11	miRNA change
	37126044	TT	13	TFBS change
	37126067	GG	34	miRNA change TFBS change
	37142283	AA	71	miRNA change TFBS change
	37187537	TT	30	INTRONIC
	37188268	CC	67	INTRONIC
	37196342	GG	12	INTRONIC
	37200097	GG	48	INTRONIC
	37201457	CC	15	INTRONIC
	37205397	CC	34	INTRONIC
	37205451	TT	19	INTRONIC
	37206251	GG	15	INTRONIC
	37215701	GG	65	INTRONIC
	37219280	CC	33	INTRONIC
	37219351	CC	14	INTRONIC
	37225007	AA	97	INTRONIC
	37225286	TT	13	INTRONIC
	37226206	AA	10	INTRONIC
	37226855	CC	16	INTRONIC
	37228813	CC	11	INTRONIC
	37229626	GG	19	INTRONIC
	37233185	TT	13	INTRONIC
	37234742	AA	31	INTRONIC
	37236643	AA	15	INTRONIC
	37247325	GG	15	INTRONIC
	37250978	CC	80	INTRONIC
	37265749	GG	40	INTRONIC
	37266392	TT	13	INTRONIC
	37284795	TT	13	TFBS change
	37287964	GG	37	miRNA change TFBS change
	37290783	CC	12	TFBS change
	37292365	AA	12	miRNA change
	37294131	CC	20	miRNA change
	37343613	TT	12	miRNA change TFBS change
	37363999	GG	30	TFBS change
	37364553	AA	27	TFBS change
	37452057	GG	10	INTRONIC
	37452124	AA	22	INTRONIC
	37458708	CC	12	INTRONIC
	37458865	AA	10	INTRONIC
	37458871	AA	16	INTRONIC
	37459537	GG	40	INTRONIC
	37472355	GG	16	INTRONIC
	37472611	AA	47	INTRONIC
	37476579	TT	30	INTRONIC
	37477693	CC	12	INTRONIC
	37478601	GG	26	INTRONIC
	37480959	CC	30	INTRONIC
	37482457	GG	15	INTRONIC
	37491502	GG	33	INTRONIC
	37492736	AA	21	INTRONIC
	37493967	TT	10	INTRONIC
	37494406	AA	72	INTRONIC
	37494485	CC	14	INTRONIC
	37498910	TT	12	INTRONIC
	37499390	GG	10	INTRONIC
	37502647	GG	27	INTRONIC
	37506729	AA	20	INTRONIC
	37516868	AA	10	INTRONIC
	37521109	TT	87	INTRONIC
	37521868	CC	97	INTRONIC
	37522527	TT	25	INTRONIC
	37529231	TT	13	INTRONIC
	37529548	TT	14	INTRONIC
	37536096	AA	12	INTRONIC
	37538376	AA	16	INTRONIC
	37544162	TT	17	INTRONIC
	37555733	GG	12	INTRONIC
	37555819	CC	13	INTRONIC
	37558543	CC	13	INTRONIC
	37559687	TT	11	INTRONIC
	37561820	CC	27	INTRONIC
	37564422	AA	14	INTRONIC
	37566512	CC	12	INTRONIC
	37568606	CC	15	INTRONIC
	37570668	CC	13	INTRONIC
	37574392	CC	43	INTRONIC
	37574975	GG	10	INTRONIC
	37579188	TT	24	INTRONIC
	37582007	TT	19	INTRONIC
	37582812	GG	16	INTRONIC
	37585611	CC	45	INTRONIC
	37594353	CC	94	INTRONIC
	37598499	CC	57	INTRONIC
	37605138	AA	10	INTRONIC
	37605139	TT	11	INTRONIC
	37605730	AA	15	INTRONIC
	37606719	GG	10	INTRONIC
	37627824	TT	10	INTRONIC
	37658166	TT	11	INTRONIC
	37710073	CC	29	TFBS change
	37714749	CC	26	TFBS change
	37715890	GG	94	TFBS change
	37738150	AA	31	miRNA change
	37744880	GG	16	miRNA change TFBS change
	37750938	AA	10	miRNA change TFBS change
	37754220	GG	17	miRNA change
	37754259	AA	12	miRNA change
	37755267	AA	15	TFBS change
	37770210	TT	77	miRNA change
	37824729	CC	39	TFBS change
	37849581	AA	34	miRNA change
	37856808	GG	11	TSS PROXIMITY miRNA change
	37856942	AA	20	TSS PROXIMITY
	37868611	TT	68	TSS PROXIMITY
	37871156	GG	55	TSS PROXIMITY miRNA change TFBS change
*	37871992	GG	68	TSS PROXIMITY TES PROXIMITY miRNA change
	37872738	AA	28	TSS PROXIMITY miRNA change TFBS change
	37873638	AA	57	TSS PROXIMITY miRNA change
	37875270	GG	18	TSS PROXIMITY
	37877147	AA	19	TSS PROXIMITY miRNA change TFBS change
	37882662	AA	12	TSS PROXIMITY TFBS change
	37888494	AA	13	TSS PROXIMITY TFBS change
	37891712	AA	14	TSS PROXIMITY
	37940165	GG	16	TSS PROXIMITY TFBS change
	37940954	TT	17	TSS PROXIMITY
	37941830	AA	17	TSS PROXIMITY
	37944067	TT	12	TSS PROXIMITY TFBS change
	37948797	CC	14	TSS PROXIMITY TFBS change
	37951161	AA	20	TSS PROXIMITY TFBS change
	37951388	AA	85	TSS PROXIMITY
	37952197	TT	12	TSS PROXIMITY miRNA change
	37953673	TT	12	TSS PROXIMITY TES PROXIMITY
	37956685	AA	30	TSS PROXIMITY TFBS change
	37957180	AA	15	TSS PROXIMITY
	37958884	TT	76	TSS PROXIMITY
	37959750	CC	28	TSS PROXIMITY miRNA change
	37960878	CC	57	TSS PROXIMITY
	37965635	GG	37	TSS PROXIMITY
	37968559	GG	28	TSS PROXIMITY
	37969143	TT	10	TSS PROXIMITY
	37969840	GG	20	TSS PROXIMITY
	37970147	TT	21	TSS PROXIMITY
	37972395	AA	25	TSS PROXIMITY
	37973986	AA	16	TSS PROXIMITY TFBS change
	37978265	GG	20	miRNA change TFBS change
	37981482	GG	10	TFBS change
	37983410	TT	20	miRNA change TFBS change
	37987832	TT	64	TFBS change
	37996273	CC	28	TFBS change
	38006534	GG	47	TFBS change
	38006866	AA	42	miRNA change TFBS change ncRNA PROXIMITY
	38017695	GG	34	miRNA change TFBS change
	38029263	TT	82	TFBS change
	38031354	TT	40	miRNA change
	38060116	AA	15	TFBS change
	38060996	CC	33	miRNA change
	38065657	AA	23	TFBS change
	38066137	CC	10	TFBS change
	38068479	AA	78	miRNA change
	38073660	AA	15	miRNA change
	38075216	TT	54	miRNA change TFBS change
	38111851	AA	10	miRNA change
	38116133	CC	14	TFBS change
	38147726	AA	15	miRNA change
	38153973	TT	26	TFBS change
	38161074	AA	19	miRNA change
	38162115	GG	51	miRNA change
	38164334	GG	34	TFBS change
	38166479	TT	63	TFBS change
	38183881	AA	15	TSS PROXIMITY miRNA change
	38183927	GG	17	TSS PROXIMITY miRNA change
	38191074	CC	34	TSS PROXIMITY miRNA change
	38192878	CC	23	TSS PROXIMITY miRNA change TFBS change
	38195435	TT	46	INTRONIC
	38197486	CC	27	INTRONIC
	38199290	AA	10	INTRONIC
	38199291	AA	10	INTRONIC
	38207212	CC	17	INTRONIC
	38207509	AA	88	INTRONIC
	38210134	CC	19	INTRONIC
	38215979	GG	12	INTRONIC
	38219313	TT	26	INTRONIC
	38227102	TT	21	INTRONIC
	38227103	GG	23	INTRONIC
	38227465	TT	16	INTRONIC
	38228487	GG	21	INTRONIC
	38229535	TT	75	INTRONIC
	38236011	GG	39	INTRONIC
	38242115	AA	71	INTRONIC
	38247538	GG	25	INTRONIC
	38247787	CC	14	INTRONIC
	38247898	TT	40	INTRONIC
	38255608	CC	13	INTRONIC
	38255626	GG	10	INTRONIC
	38258165	CC	13	INTRONIC
	38264424	TT	30	INTRONIC
	38272464	AA	31	INTRONIC
	38277482	CC	95	INTRONIC
	38297034	GG	11	INTRONIC
	38297707	AA	71	INTRONIC
	38298904	TT	27	INTRONIC
	38299362	TT	14	INTRONIC
	38303738	TT	16	INTRONIC
	38305559	AA	27	INTRONIC
	38305634	TT	12	INTRONIC
	38309469	GG	12	INTRONIC
	38310641	CC	15	INTRONIC
	38311049	TT	55	INTRONIC
	38312454	GG	36	INTRONIC
	38314468	GG	13	INTRONIC
	38315461	TT	24	INTRONIC
	38316970	AA	33	INTRONIC
	38319784	GG	98	INTRONIC
	38319865	TT	56	INTRONIC
	38320085	GG	32	INTRONIC
	38321560	TT	11	INTRONIC
	38321904	CC	29	INTRONIC
	38325057	GG	32	INTRONIC
	38329848	AA	11	INTRONIC
	38340130	AA	22	INTRONIC
	38340847	GG	32	INTRONIC
	38344903	GG	27	INTRONIC
	38344904	TT	26	INTRONIC
	38348649	AA	31	INTRONIC
	38377727	CC	22	miRNA change
	38378296	GG	13	miRNA change
	38378319	AA	18	miRNA change
	38382993	GG	36	miRNA change
	38383075	TT	81	miRNA change
	38396494	TT	13	TSS PROXIMITY
	38397559	TT	16	TSS PROXIMITY TFBS change
	38397797	AA	10	TSS PROXIMITY miRNA change TFBS change
	38400970	TT	84	TSS PROXIMITY
	38402819	TT	23	TSS PROXIMITY TFBS change
	38402956	GG	12	TSS PROXIMITY miRNA change TFBS change
	38412468	GG	22	TSS PROXIMITY miRNA change TFBS change
	38412701	TT	17	TSS PROXIMITY miRNA change
	38430308	CC	96	TSS PROXIMITY miRNA change
	38433536	CC	29	TSS PROXIMITY miRNA change TFBS change
	38443159	TT	12	TSS PROXIMITY TFBS change
	38448533	CC	14	INTRONIC
	38448582	CC	13	INTRONIC
	38452271	CC	99	TSS PROXIMITY
	38456065	CC	35	TSS PROXIMITY miRNA change
	38456369	TT	16	TSS PROXIMITY miRNA change
	38457028	TT	53	TSS PROXIMITY
	38457187	AA	24	TSS PROXIMITY miRNA change
	38464347	TT	24	TSS PROXIMITY TES PROXIMITY miRNA change TFBS change
	38466720	AA	36	TSS PROXIMITY miRNA change
	38466749	GG	93	TSS PROXIMITY miRNA change
	38470356	AA	12	TSS PROXIMITY miRNA change
	38490914	CC	15	INTRONIC
	38491832	AA	38	INTRONIC
	38507808	AA	22	TSS PROXIMITY
	38508991	CC	11	TSS PROXIMITY miRNA change
*	38513135	CC	41	EXONIC
	38513883	TT	17	INTRONIC
	38514699	TT	40	INTRONIC
*	38514745	TT	34	EXONIC
	38519825	CC	80	INTRONIC

*The table shows SNPs identified during resequencing within the CFA12: 36,750,205–38,524,449 genomic region for which the case is homozygous. SNPs that are homozygous in the case but without any further comments have been removed from the table. Further SNPs are only included in the table if Cons score ≥10. A paper describing details of the functional prediction of the SNPS is in preparation. ^a^SNPs selected for TaqMan genotyping. ^b^Position according on CFA12 according to Ensembl *Canis familiaris* version 64.2. ^c^Genotype from resequencing data. ^d^Conservation in dog, human, mouse, and rat are computed by UCSC’s phastCons and multiplied by 100. ^e^Each SNP was annotated based on the following features: EXONIC, the SNP is in an annotated exon; INTRONIC, the SNP is in an annotated intron; TSS PROXIMITY, the SNP is close to a transcription start site; TES PROXIMITY, the SNP is close to a transcription end site; miRNA change, the SNP changes the predicted binding of miRNA; TFBS change, the SNP changes the predicted binding of a transcription factor; ncRNA, the SNP is in or near a predicted ncRNA*.

**Significance of 37871992 is 1.4 ×10^−7^; 38513135 is 0.00087; 38514745 is 0.01707*.

**Table A2:** **Haplotype substitution effects on linear and logistic scales for disc calcification scored as binary case/control**.

Haplotypes	Haplotype window	Linear model	Logistic model^1^
	Hap 1	*R*^2^ = 73%	RMD^3^ = 0.64
α_0_ = H4		−0.49	−8.53
H1		0.71	5.93
H2		0.20	−5.28*^ns^*
H3		−0.22*^ns^*	−8.40*^ns^*
	Hap 2	*R*^2^ = 73%	RMD = 0.64
α_0_ = H3		−0.36	−8.91
H1		0.63	6.14
H2		−0.27*^ns^*	−8.24*^ns^*
	Hap 3	*R*^2^ = 76%	RMD = 0.46
α_0_ = H4		−0.41	−22.97
H1		0.66	15.52
H2		0.39	12.06*^ns^*
H3		0.03*^ns^*	1.056*^ns^*
	Hap 4	*R*^2^ = 51%	RMD = 0.92
α_0_ = H3		−0.41	−19.36
H1		0.63	11.08
H2		0.36	9.26
	Hap 5	*R*^2^ = 68%	RMD = 0.75
α_0_ = H3		−0.31	−7.03
H1		0.61	5.28
H2		−0.33	−6.13*^ns^*
H3		−0.31*^ns^*	−9.25*^ns^*
	Hap 6	*R*^2^ = 63%	RMD = 0.85
α_0_ = H2		−0.31	−6.82
H1		0.60	4.73
	Hap 7	*R*^2^ = 63%	RMD = 0.82
α_0_ = H3		−0.32	−7.41
H1		0.61	5.35
H2		0.38	2.77*^ns^*
	Hap 8	*R*^2^ = 63%	RMD = 0.85
α_0_ = H2		−0.31	−6.82
H1		0.59	4.73
	Hap 9	*R*^2^ = 62%	RMD = 0.76
α_0_ = H4		−0.40	−19.45
H1		0.64	11.01
H2		0.11*^ns^*	0.94*^ns^*
H3		0.14	7.68*^ns^*

*Tests of association of haplotypes (coded as H1, H2, etc.) from CFA12: 36,750,205–38,524,449; those haplotype effects that were not significant at *P* < 0.01 are marked with superscript *ns* in each haplotype window. ^1^Estimates on logit scale can be back transformed to probability of observing a case or control by π = 1/(1 + *e*^−η^) where η is an estimate of haplotype effects. ^2^*R*^2^, percent variability in the data set that is accounted for by the fitted haplotype block model; ^3^RMD, residual mean deviance is an indicator for goodness of fit (lower is better)*.

**Table A3:** **Specification of the primers and probes in the SNP genotyping assays**.

SNP location	Forward primer; reverse primer	Probes labeled with VIC^®^/FAM^™^ fluorescent dye
37,871,992	F: TTCGAATTTGAAGCTAAGACTGCTAGAA; R: AACCGCCCGGGCTT	VIC: CCCTCTC**C**GCCCCC; FAM: CCTCTC**G**GCCCCC
38,513,135	F: AGAGCAGAATTTATCCAGTTCCTTTCG; R: AACAGGAAAGATTGCTTAAAACTAATGAAGT	VIC: TTTCCAAACTT**T**GTTTTCA; FAM: CCAAACTT**C**GTTTTCA
38,514,745	F: ACCTGCAACATTTTACTCCATCACTT; R: GACCTTTTAAAAAGTCATGGGCAGT	VIC: TCACAGCA**A**GTTTTAG; FAM: TCACAGCA**T**GTTTTAG

*SNP location in base pairs; F, forward primer; R, reverse primer; VIC, VIC ^®^ fluorescent dye, FAM, FAM^™^ fluorescent dye*.

**Table A4:** **Top allelic association hits in the GWAS on disc calcification in 33 wire-haired cases and 28 wire-haired controls, sorted by genomic position**.

Haplotype window	Canine SNP	Chr	Pos	*P*_genome_	A_R_/A_NR_
Hap 1	BICF2P1218920	12	36750205	0.02646	A/T
Hap 1	BICF2P909271	12	36756197	0.02646	G/A
Hap 1	TIGRP2P163331	12	36770550	0.02646	G/T
Hap 1	BICF2S23234423	12	36909311	3.0E-5	T/C
Hap 2	TIGRP2P163344	12	37056901	3.0E-5	T/C
Hap 2	BICF2P1304914	12	37079212	3.0E-5	G/A
Hap 2	BICF2P211642	12	37099752	3.0E-5	A/C
Hap 2	BICF2P979506	12	37119065	3.0E-5	G/A
Hap 3	BICF2P16177	12	37123193	3.0E-5	T/G
Hap 3	BICF2P825805	12	37134630	3.0E-5	A/C
Hap 3	BICF2S23242450	12	37480959	1.0E-5	C/A
Hap 3	BICF2S23240823	12	37494845	8.7E-4	A/G
Hap 4	BICF2S23023749	12	37710073	0.00916	C/T
Hap 4	BICF2S23043206	12	37733597	0.00916	T/C
Hap 4	G745F34S150	12	37806613	0.00916	G/A
Hap 4	BICF2P717725	12	37826314	0.00392	T/G
Hap 5	BICF2P1197203	12	37847222	0.00916	G/A
Hap 5	TIGRP2P163387	12	37859396	0.00916	C/T
Hap 5	BICF2S23632751	12	37899159	9.0E-5	T/C
Hap 5	TIGRP2P163398	12	37944067	9.0E-5	T/C
Hap 6	BICF2P1304952	12	37958884	9.0E-5	T/G
Hap 6	TIGRP2P163406	12	37980930	9.0E-5	T/G
Hap 6	BICF2P478656	12	38003121	9.0E-5	C/T
Hap 6	BICF2P371497	12	38015502	9.0E-5	T/C
Hap 7	BICF2P31931	12	38022379	9.0E-5	C/A
Hap 7	BICF2S22962067	12	38042875	9.0E-5	T/C
Hap 7	BICF2P462046	12	38064467	0.00922	G/A
Hap 7	BICF2P1309489	12	38072703	9.0E-5	T/C
Hap 8	BICF2P114736	12	38079788	9.0E-5	A/C
Hap 8	BICF2P1354926	12	38182743	9.0E-5	G/A
Hap 8	BICF2P320495	12	38202857	9.0E-5	G/A
Hap 8	BICF2P1089702	12	38229535	9.0E-5	T/A
Hap 9	TIGRP2P163437	12	38264121	9.0E-5	A/G
Hap 9	BICF2S23241475	12	38348649	0.01039	A/C
Hap 9	TIGRP2P163478	12	38507494	0.00333	T/C
Hap 9	BICF2P1077702	12	38524449	0.00333	G/T

*Chr, chromosome; Pos, physical position; *P*_genome_, *p*-value corrected for multiple testing by permutation; A_R_, risk allele; A_NR_, non-risk allele*.

## Abbreviations

BLUP, best linear unbiased prediction; CFA, canine chromosome; DDC, Danish Dachshund Club; GLM, generalized linear model; GWAS, genome-wide association studies; LD, linkage disequilibrium; ncRNAs, non-coding RNAs; qPCR, quantitative fluorescence PCR; RMD, residual mean deviance; *R*^2^, coefficient of determination; UTRs, untranslated regions.
